# The Parkinson's Disease Associated LRRK2 Exhibits Weaker *In Vitro* Phosphorylation of 4E-BP Compared to Autophosphorylation

**DOI:** 10.1371/journal.pone.0008730

**Published:** 2010-01-15

**Authors:** Azad Kumar, Elisa Greggio, Alexandra Beilina, Alice Kaganovich, Diane Chan, Jean-Marc Taymans, Benjamin Wolozin, Mark R. Cookson

**Affiliations:** 1 Cell Biology and Gene Expression Unit, Laboratory of Neurogenetics, National Institute on Aging, National Institutes of Health, Bethesda, Maryland, United States of America; 2 Departments of Pharmacology and Neurology, Boston University School of Medicine, Boston, Massachusetts, United States of America; 3 Laboratory for Neurobiology and Gene Therapy, Division of Molecular Medicine, Department of Molecular and Cellular Medicine, Katholieke Universiteit Leuven, Leuven, Belgium; University of Melbourne, Australia

## Abstract

Mutations in the gene encoding *Leucine-rich repeat kinase 2* (*LRRK2*) are the most common cause of inherited Parkinson's disease (PD). LRRK2 is a multi-domain protein kinase containing a central catalytic core and a number of protein-protein interaction domains. An important step forward in the understanding of both the biology and the pathology of LRRK2 would be achieved by identification of its authentic physiological substrates. In the present study we examined phosphorylation of 4E-BP (eukaryotic initiation factor 4E (eIF4E)-binding protein), a recently proposed substrate for LRRKs. We found that LRRK2 is capable of phosphorylating 4E-BP *in vitro*. The PD related LRRK2-G2019S mutant was ∼2 fold more active than wild type protein. However, LRRK2 autophosphorylation was stronger than 4E-BP phosphorylation under conditions of molar excess of 4E-BP to LRRK2. We also tested three other kinases (STK3, MAPK14/p38α and DAPK2) and found that MAPK14/p38α could efficiently phosphorylate 4E-BP at the same site as LRRK2 *in vitro*. Finally, we did not see changes in 4E-BP phosphorylation levels using inducible expression of LRRK2 in HEK cell lines. We also found that MAPK14/p38α phosphorylates 4E-BP in transient overexpression experiments whereas LRRK2 did not. We suggest that increased 4E-BP phosphorylation reported in some systems may be related to p38-mediated cell stress rather than direct LRRK2 activity. Overall, our results suggest that 4E-BP is a relatively poor direct substrate for LRRK2.

## Introduction

Parkinson's disease (PD) is a common and currently incurable neurodegenerative movement disorder affecting approximately 1–2% of the population over 65 years of age. Clinically, PD is characterized by a movement disorder that includes resting-tremor, muscular rigidity, and bradykinesia, as well as non-motor symptoms. Neuropathologically, selective loss of dopaminergic (DA) neurons in the *substantia nigra compacta region* and formation of lewy body in surviving neurons are two hallmarks of PD patient brains [1].

Dominant mutations in the gene *LRRK2* (*Leucine-rich repeat kinase 2*) are the most common cause of inherited PD [2]–[4], and most cases of *LRRK2*-related PD are pathologically similar to sporadic disease. *LRRK2* encodes for a large, 286 kDa, multidomain protein [5] consisting of a catalytic core and a number of putative protein-protein interaction domains, including N-terminal ankyrin repeats, a leucine-rich repeat region, and a C-terminal WD40 domain. The catalytic core consists of a GTPase/ROC (Ras Of Complex protein) domain, followed by a COR (C-terminus Of ROC) domain and a serine/threonine kinase domain. Mutations that segregate with PD are mainly clustered within the catalytic core, suggesting that alteration of the enzymatic functions may be correlated with pathology. There are mutations in the kinase domain (G2019S and I2020T) and in the ROC-COR bi-domain (R1441C and Y1699C) that may affect either kinase or GTPase activity [5]–[9].

The most prevalent LRRK2 mutation, G2019S [10], lies within the Mg^+2^-binding motif (DYG) of the kinase domain, and has been shown to increase the kinase activity of LRKK2 [8], both in heterologous and autophosphorylation assays [10]–[15]. However, whether other mutations in LRRK2 affect kinase activity is controversial [15]. A caveat about several published studies is that autophosphorylation was used to measure kinase activity. Although such assays can be helpful *in vitro*, it is unclear whether autophosphorylation is physiologically relevant. An alternative to autophosphorylation as an assay for kinase activity is to use generic substrates such as myelin basic protein (MBP) [10]–[12], which generally give similar results to autophosphorylation.

Several heterologous LRRK2 substrates have been suggested for LRRK2 including moesin [13], β-tubulin [16] and MAPKKK substrates MKK3/6 or MKK4/7 [17]. Whether any of these substrates are physiologically relevant is currently unclear.

Recently Imai et al. reported 4E-BP as a potential substrate of LRRK2 [18]. 4E-BP is an interactor of the eukaryotic protein translation initiation factor eIF4E, which in turn binds to capped mRNA species, promoting their translation. Binding of 4E-BP to eIF4E prevents the latter being active and, therefore, 4E-BP is a repressor of protein translation. Oxidative stress and other stimuli that impact protein translation affect phosphorylation of 4E-BP. Imai et al. proposed that LRRK2 modulates this system by phosphorylating 4E-BP at a specific site (T37/T46), which then acts as a stimulus for further phosphorylation by other kinases at secondary sites including S65/S70. There was a modest decrease in phosphorylation of 4E-BP T37/T46 and S65 when LRRK2 levels were knocked down with RNAi. Overexpression of 4E-BP rescued the effects of LRRK mutants *in vivo* using *Drosophila* models. Similarly, Tain et al have shown that phospho-4E-BP levels are decreased in a homozygous knockout model of *drosophila* LRRK [19]. Collectively these data are supportive of 4E-BP being a substrate for LRRK2 or its *Drosophila* homologue, dLRRK. However, the kinetics of the phosphorylation reaction have not yet been reported. In the present study we investigated the kinetic properties of 4E-BP phosphorylation by LRRK2 compared to autophosphorylation activity and we used a LRRK2 inducible cellular model as well as transient expression in HEK cells to measure the effect of LRRK2 expression on 4E-BP activation at T37/T46.

## Results

### *In Vitro* Kinase Assay

**Figure 1:**
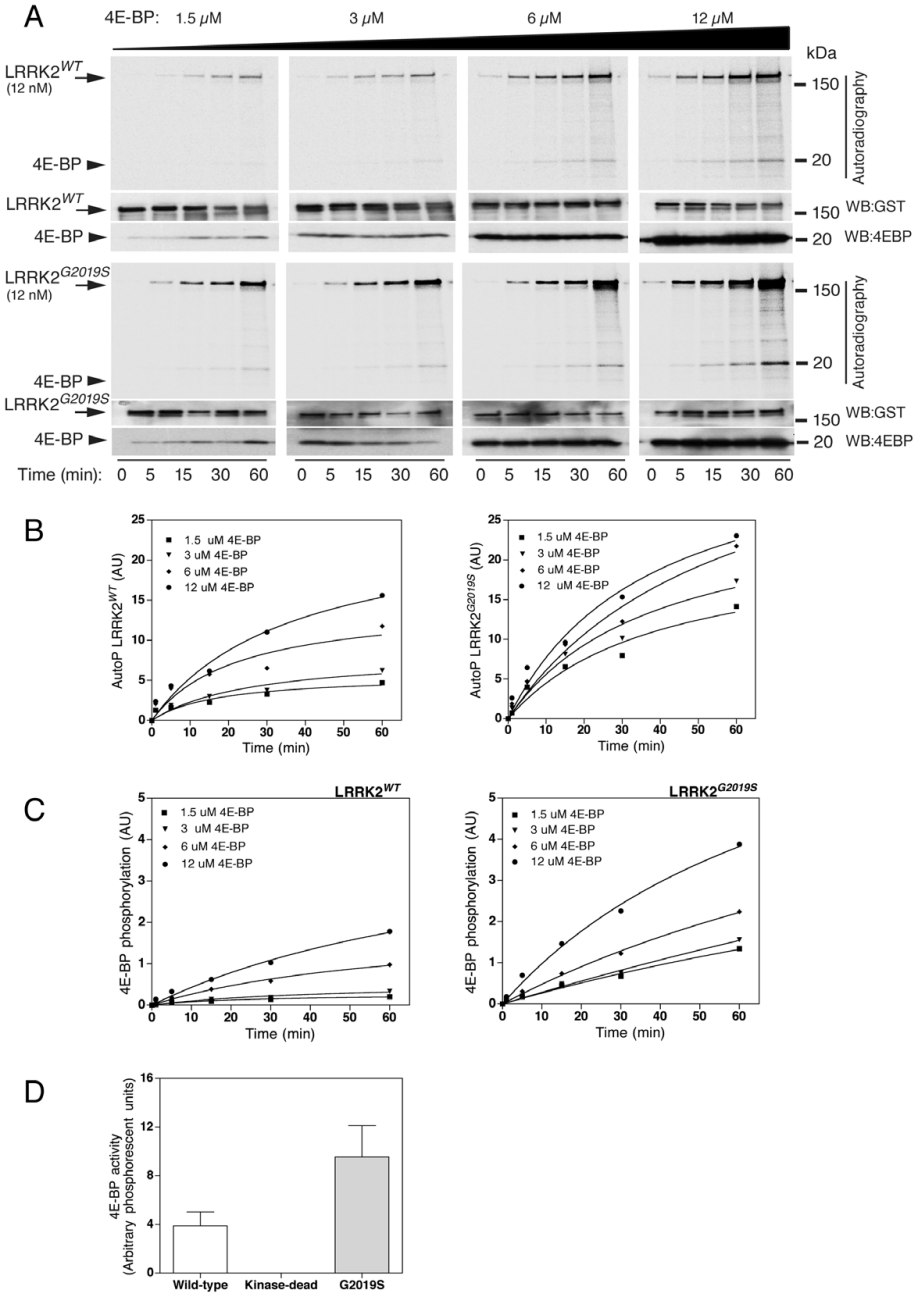
Phosphorylation of 4E-BP by LRRK2 *in vitro*. (A) Time-course catalytic assays were performed for GST-LRRK2 wild-type (upper panel) or GST-LRRK2-G2019S (lower panels) by measuring the incorporation of [γ^33^P] ATP at different time points (0, 5, 15, 30 and 60 mins) and at different concentrations of 4E-BP (1.5, 3, 6 and 12 µM), respectively, followed by SDS-PAGE and autoradiography. (B) Autophosphorylation of LRRK2-wild type (left graph) and G2019S (right graph) were plotted as a function of time. (C) Phosphorylation of 4E-BP by LRRK2-wild type (left graph) and G2019S (right graph) were plotted as a function of time. (D) Results of the catalytic assays were derived by calculating the reaction rate of each assay at different 4E-BP concentrations (1.5, 3, 6 and 12 µM). For all experiments in A–D, each point is the average of three independent experiments and error bars indicate the SEM.

We first examined 4E-BP phosphorylation by LRRK2-wild type and LRRK2-G2019S *in vitro* (Figure 1). We used recombinant protein expressed in insect cells containing amino acids 970–2527 of human LRRK2 that has been previously shown to be highly active [14] rather than immunoprecipitated LRRK2 from mammalian cells as the latter may contain contaminant kinases. We confirmed [18] that LRRK2 variants were able to phosphorylate 4E-BP and that the LRRK2-G2019S mutant increased 4E-BP phosphorylation compared to wild-type proteins. As a negative control, a kinase dead LRRK2 (D1994A) mutant had a negligible activity towards 4E-BP phosphorylation under the same conditions (data not shown).

After confirming that 4E-BP is an *in vitro* substrate for LRRK2, we assessed the kinetic catalytic activity of LRRK2-wild type, LRRK2-kinase dead and LRRK2-G2019S toward 4E-BP. Increasing concentrations of 4E-BP (1.5, 3, 6 and 12 µM) were incubated with 12 nM of LRRK2, and the incorporation of ^33^P over the time-course of reaction was measured (Figure 1A, C and D). The efficiency of 4E-BP phosphorylation was almost two times higher for LRRK2-G2019S (11.9±0.66, arbitrary phosphorescence units) compared to LRRK2-wild type (5.1±0.45, arbitrary phosphorescence units), and again kinase dead LRRK2 was inactive (Table 1). However, K_m_ for 4E-BP was similar for both wild type and LRRK2-G2019S (2.69±0.64 µM and 2.87±0.45 µM, respectively).

**Table 1:** V_max_ and K_m_ for LRRK2 and MAPK14/P38α phosphorylation of 4E-BP *in vitro*.

Kinase	WT LRRK2	G2019S LRRK2	MAPK14/P38α
V_max_ (arbitrary phosphorescence unit)	5.1±0.45	11.9±0.66	102±8.14
K_m_(µM)	2.69±0.641	2.87±0.452	2.56±0.592

Each data point is calculated V_max_ and K_m_ values (±SEM) for assays carried out in triplicate.

Interestingly, under the same conditions incorporation of radioactivity into LRRK2 was substantially higher than 4E-BP phosphorylation, despite LRRK2 being present at a much lower concentration (∼12 nM LRRK2 *versus* 1.5 to 12 µM 4E-BP), which is apparent on examination of a single exposure with both reactions (Figure 1A). Quantitation demonstrated that for 12 nM LRRK2 and 12 µM 4E-BP, almost three times as much radioactivity is incorporated into LRRK2 (Figure 1B and C). As for 4E-BP phosphorylation, G2019S increased autophosphorylation activity by about two-fold (Figure 1B) and D1994A abolished activity (data not shown).

**Figure 2:**
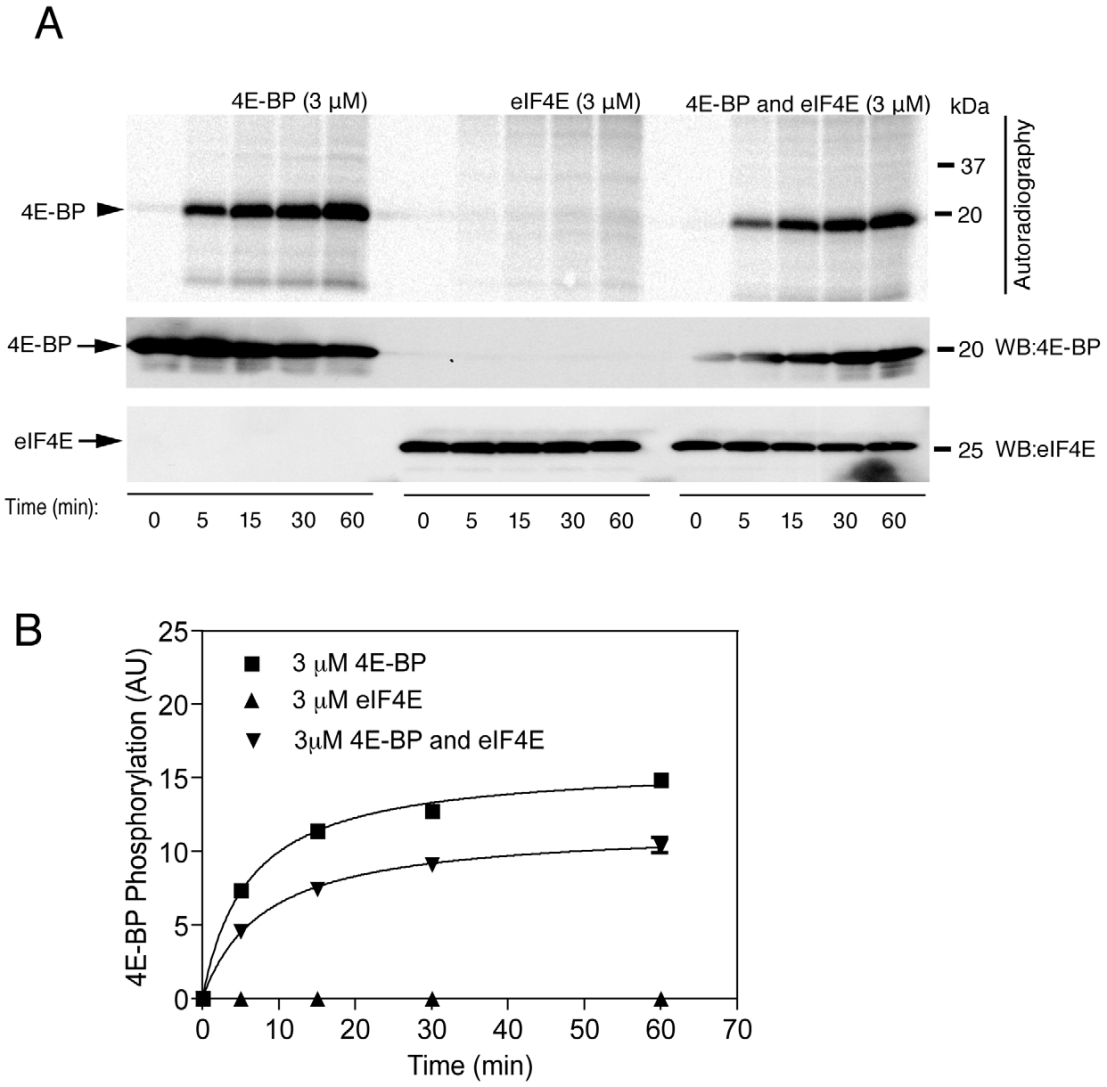
Effect of 4E-BP and eIF4E complex on phosphorylation of 4E-BP by LRRK2 *in vitro*. (A) Time-course assays were performed for LRRK2 wild-type (upper panel) by measuring the incorporation of [γ^33^P] ATP at different time points (0, 5, 15, 30 and 60 mins) and at 3 µM concentrations of 4E-BP (upper left panel), 3 µM concentrations of eIF4E (upper middle panel) and 3 µM concentrations of 4E-BP and 3 µM concentrations of eIF4E (upper right panel), respectively, followed by SDS-PAGE and autoradiography. (B) Phosphorylation of 4E-BP by LRRK2-wild type were plotted as a function of time.

Prior studies have suggested that phosphorylation of 4E-BP can be affected by the presence of its binding partner eIF4E [20]. We compared kinase activity of wild type LRRK2 towards 4E-BP alone or in an equimolar complex with eIF4E (Figure 2A and B). We found that the presence of eIF4E partially blocked 4E-BP phosphorylation but did not prevent phosphorylation of 4E-BP entirely. No evidence of phosphorylation of eIF4E was found. These results suggest that LRRK2 can phosphorylate 4E-BP weakly even in the presence of eIF4E.

**Figure 3:**
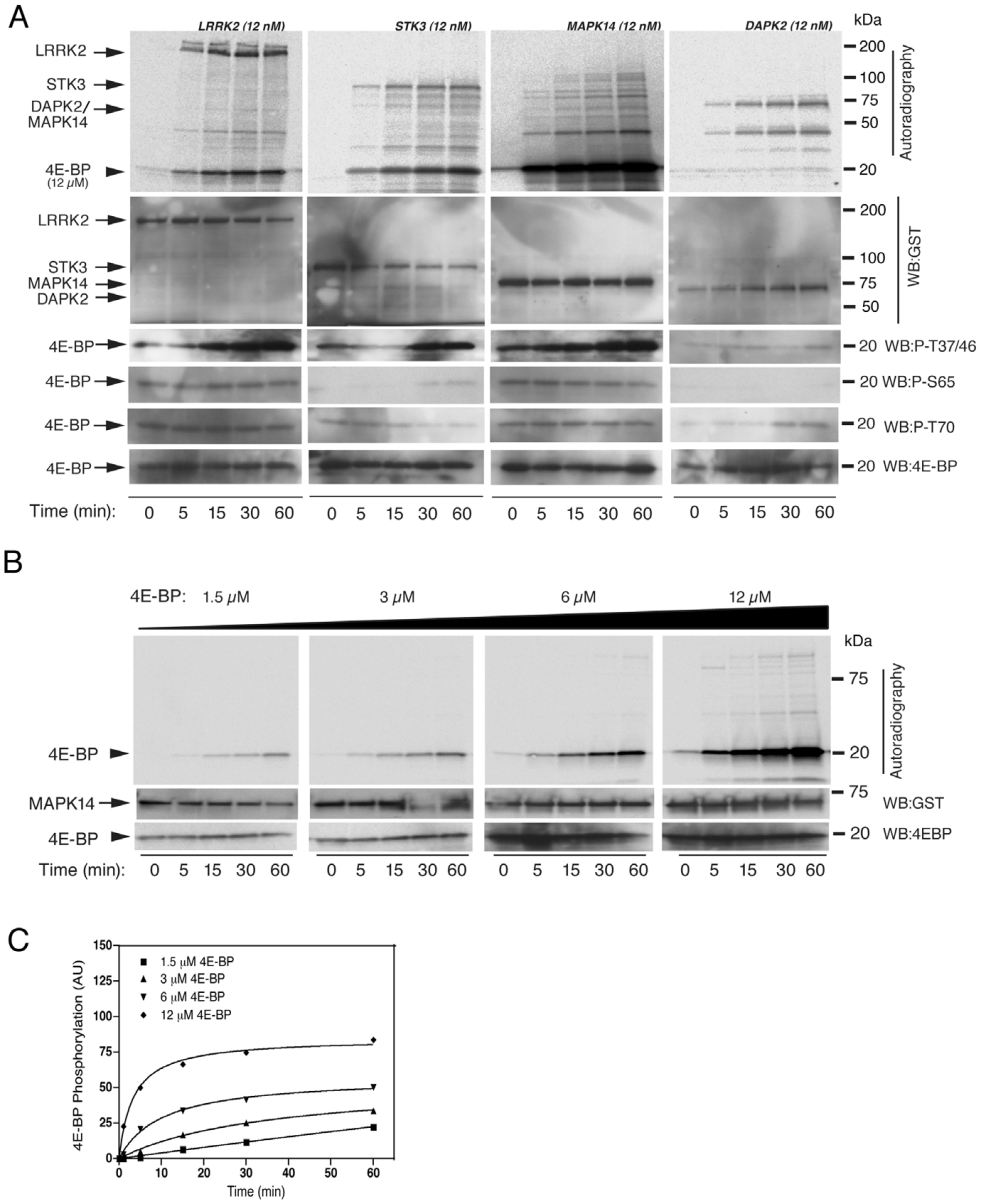
Kinases other than LRRK2 can also phosphorylate 4E-BP. (A) Kinase assays were performed for LRRK2, STK3, MAPK14//P38α and DAPK2 by measuring the incorporation of [γ^33^P] ATP at different time points (0, 5, 15, 30 and 60 mins) and at 12 µM concentrations of 4E-BP, followed by SDS-PAGE and autoradiography. (B) Time-course catalytic assays were performed for MAPK14/P38α by measuring the incorporation of [γ^33^P] ATP at different time points (0, 5, 15, 30 and 60 mins) and at different concentrations of 4E-BP (1.5, 3, 6 and 12 µM) respectively, followed by SDS-PAGE and autoradiography. (C) 4E-BP(T37/46) phosphorylation is plotted as a function of time.

We next considered whether kinases other than LRRK2 could mediate the same reaction picking three kinases from different families in the kinome (Figure 3). Under conditions similar to the LRRK2 reactions, MAPK14/p38α lacks autokinase activity but STK3 and DAPK2 could autophosphorylate. We found that MAPK14/p38α phosphorylated 4E-BP at a much higher rate (Figure 3A and B) compared to STK3 whereas DAPK2 showed no reactivity towards 4E-BP. These results suggest that 4E-BP can be phosphorylated by several different kinases *in vitro*. We also noted that the putative LRRK2 site recognized by antibodies against T37/T46 were also phosphorylated by MAPK14/p38α. The efficiency of 4E-BP phosphorylation was almost twenty times higher for MAPK14/p38α (102±8.14, arbitrary phosphorescence units) compared to LRRK2-wild type (5.1±0.45, arbitrary phosphorescence units). However, K_m_ for 4E-BP was similar for both wild type LRRK2 and MAPK14/p38α (2.69±0.64 µM and 2.56±0.59 µM, respectively) (Table 1).

### Stoichiometry of LRRK2 Autophosphorylation and 4E-BP Phosphorylation

To determine the stoichiometry of LRRK2 autophosphorylation compared to 4E-BP phosphorylation, 12 µM of 4E-BP was incubated with 12 nM of LRRK2, and the incorporation of ^33^P was measured by scintillation counting. We observed that 1 mole of ATP was incorporated into 70 moles of LRRK2 whereas 1 mole of ATP was incorporated into 1.58×10^3^ moles of 4E-BP after 60 minutes of reaction suggesting that LRRK2 autophosphorylation is approximately 20 times more efficient than 4E-BP phosphorylation under the same conditions. Table 2 shows the percentage ratio between the number of inorganic phosphates (Pi) incorporated by LRRK2 or 4E-BP and the total number of LRRK2 or 4E-BP molecules at different time points.

**Table 2:** Stoichiometry of 4E-BP phosphorylation and LRRK2 autophosphorylation.

Time (min)	0	5	15	30	60
**4E-BP**	0.0002±0.00003%	0.0007±0.00033%	0.0012±0.00015%	0.0015±0.00012%	0.0017±0.00026%
**LRRK2**	0.08±0.03%	0.44±0.06%	0.90±0.08%	1.24±0.20%	1.39±0.23%

Ratio between the number of inorganic phosphates (Pi) incorporated by LRRK2 or 4E-BP and the total number of LRRK2 or 4E-BP molecules at different time points. Each figure is the mean +/− for assays carried out in triplicate.

### Phosphorylation of 4E-BP by LRRK2 in Cells

**Figure 4:**
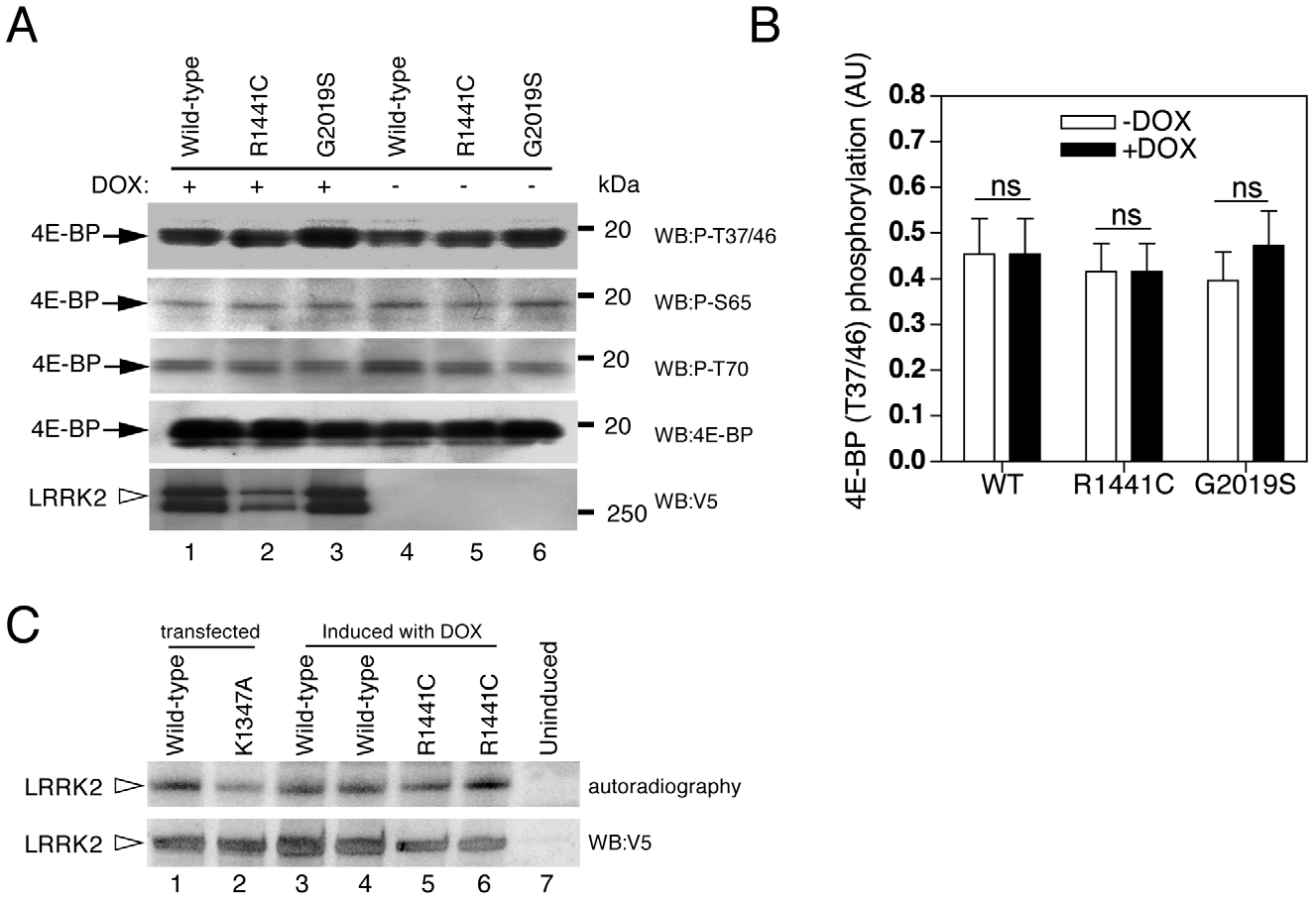
4E-BP phosphorylation in inducible HEK 293FT cells. (A) 4E-BP phosphorylation before and after induction of LRRK2 expression. The panels represent phospho-4E-BP (T37/46), phospho-4E-BP (S65), phospho-4E-BP (T70), total 4E-BP and V5-LRRK2, respectively. (B) Quantification analysis of 4E-BP phosphorylation of n = 14 independent experiments. (C) Autophosphorylation assays of LRRK2 immunoprecipitated from HEK 293FT cells. Lanes 1 and 2 are immunoprecipitated V5-tagged LRRK2 proteins transiently transfected and lanes 3–7 represent V5-LRRK2 immunoprecipitated from inducible cells.

Next we assessed the effect of expression of LRRK2 in inducible stable HEK 293FT clones. Although we were able to readily detect the phospho-4E-BP T37/T46, S65 and T70 epitope in these cells (Figure 4A), we did not see increased LRRK2 4E-BP phosphorylation upon LRRK2 expression, even when the pathological mutations G2019S or R1441C were introduced (Figure 4B). LRRK2 protein extracted and immunoprecipitated from these cells was active in kinase assays (Figure 4C).

**Figure 5:**
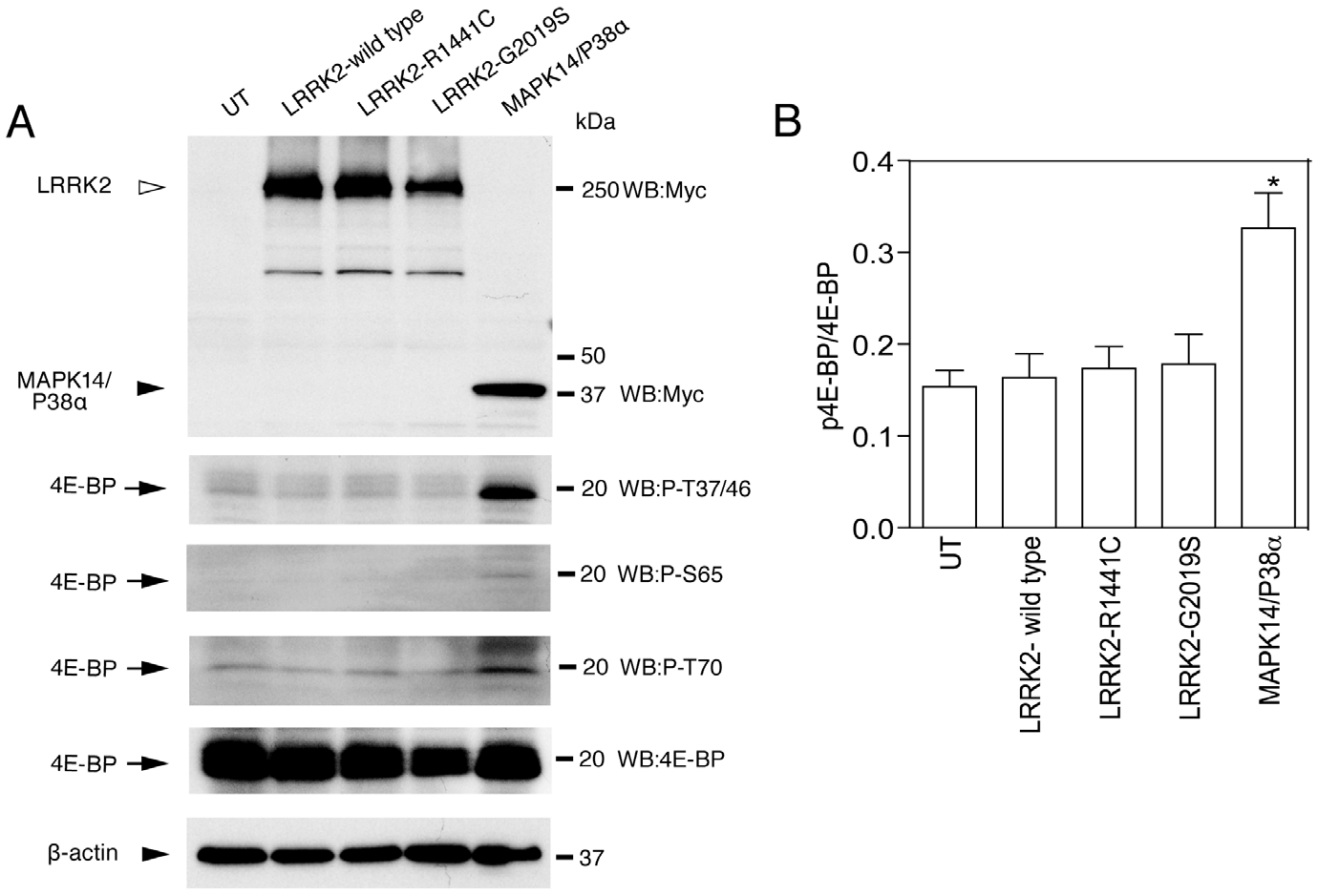
4E-BP phosphorylation in transiently transfected HEK 293FT cells. 4E-BP phosphorylation in HEK 293FT cell after transient transfection of LRRK2-wild type, LRRK2-R1441C, LRRK2-G2019S and MAPK14/P38α constructs. The panels represent myc-LRRK2 and myc-MAPK14/P38α, phospho-4E-BP (T37/46), phospho-4E-BP (S65), phospho-4E-BP (T70), total 4E-BP and β-actin, respectively. (B) Quantification of 4E-BP phosphorylation at T37/46, normalized to total 4E-BP from n = 3 samples representative of two independent experiments.

Finally we transiently overexpressed LRRK2-wild type, LRKK2-G2019S, LRRK2-R1441C and MAPK14/P38α in HEK 293FT cells. We did not detect any alteration in 4E-BP phosphorylation either by wild type or mutant LRRK2, although we found that MAPK14/P38α could phosphorylate 4E-BP under the same conditions (Figure 5A and B).

## Discussion

Although mutations in the *LRRK2* gene represent the most common cause of PD [2], [3], [21], little is known about *LRRK2*-linked molecular pathogenesis. Therefore, in the present study we have investigated the kinase activity of LRRK2 with respect to its autophosphorylation as well as phosphorylation of 4E-BP, a novel reported LRRK2 substrate. We were able to replicate some previous experiments showing that 4E-BP can be phosphorylated by LRRK2 *in vitro* but we found that this activity is weak compared to autophosphorylation. Moreover, using inducible LRRK2 cells or transient transfections, we could not detect any appreciable increased in 4E-BP phosphorylation after LRRK2 expression. However, we observed that MAPK14/p38α increases 4E-BP phosphorylation in HEK 293FT cells after transient expression, suggesting that MAPK14/P38α is a robust kinase for 4E-BP.

Prior experiments suggested that LRRK2 can phosphorylate 4E-BP *in vitro* and that I2020T, a pathogenic mutation found in the original PARK8-linked family [22], showed increased activity towards 4E-BP. We originally wished to establish whether all mutations in LRRK2 had enhanced activity towards 4E-BP, as this has been controversial for other measures of LRRK2 kinase activity. We were able to confirm that 4E-BP acts as a LRRK2 substrate *in vitro* and that 4E-BP phosphorylation is enhanced when incubated with LRKK2-G2019S, in line with multiple studies consistently showing an enhanced kinase activity for this mutation. Our kinetic analysis with 4E-BP also indicates that the G2019S mutation stimulates LRRK2 activity by increasing the catalytic V_max_ constant rather than enhancing substrate-kinase binding K_m_ affinity. This supports prior suggestions that G2019S alters processivity for LRRK2 rather than substrate binding and would be consistent with the relative ease by which enhanced activity of G2019S has been demonstrated. The G2019S mutation occurs within a highly conserved activation segment of the protein and is postulated to result in a gain of function of its kinase activity, compatible with its autosomal dominant mode of transmission [23], [24]. Conformational changes in the activation loop, which in many kinases are induced by phosphorylation, are needed to switch between the inactive and the active state of a kinase [25].

However, we also found that while LRRK2 is able to phosphorylate 4E-BP, autophosphorylation is more efficient under the conditions in which we performed these assays. This was perhaps not immediately appreciated from prior studies where the relative activities of LRRK2 autophosphorylation and LRRK2 mediated phosphorylation of 4E-BP were presented by showing each separately and in experiments where the concentration of LRRK2 is not known. However, we can facilitate a comparison between autophosphorylation and 4E-BP phosphorylation by using recombinant LRRK2 fragment of known concentration and by analyzing kinase reaction products on the same gel. Three-fold higher incorporation of ^33^P from labeled ATP into LRRK2 itself rather than 4E-BP, even when the later is present at more than a hundred-fold molar excess, demonstrates that the LRRK2 autophosphorylation reaction is much more efficient under these conditions. One limitation of this experiment is that a truncated LRRK2 version was used, as this construct is more soluble and active than full-length protein. However, in cell lines expressing full length LRRK2 that was active when isolated and tested *in vitro* for autokinase activity, we did not see any evidence of 4E-BP phosphorylation either (see below). These results were also strengthened by measuring the stoichiometry of phosphorylation for LRRK2 and 4E-BP in the same reaction, where we found that LRRK2 incorporated phosphate more efficiently than 4E-BP again despite the latter being present at molar excess.

Two further results give pause to the conclusion that 4E-BP is an authentic substrate for LRRK2. First, MAPK14/p38α and STK3 are capable of phosphorylating 4E-BP *in vitro*. As we have not performed an analysis of the complete kinome, we cannot be certain how many kinases can phosphorylate T37/T46 of 4E-BP but because other kinases can trigger phosphorylation of this site, the measurement of it in cell or tissue lysates is not a simple measure of LRRK2 activity *per se*. Second, in HEK 293FT inducible cell lines and in transiently transfected HEK 293FT cells we did not find any significant 4E-BP phosphorylation changes by wild-type LRRK2 or two different pathogenic mutants G2019S and R1441C, while MAPK14/P38α was able to efficiently phosphorylate 4E-BP in these cells.

A caveat to these assays is that we cannot prove that LRRK2 is active in these cells without a direct marker of LRRK2 phosphorylation, although we were able to extract LRKK2 and measure autophosphorylation activity *ex vivo*. Also, if 4E-BP is a substrate for several protein kinases, like MAPK14/P38α, the contribution of LRRK2 phosphorylation may not be sufficient to discriminate 4E-BP basal phosphorylation or it may require a specific stimulus. Furthermore, we determined kinetic parameters for MAPK14/p38α mediated phosphorylation of 4E-BP compared to LRRK2, and found that MAPK14/p38α is more efficient, based on its twenty times higher V_max_ value (Table 1). It is of interest that MAPK14/p38α, is involved in cell death and it is possible that its activation could also occur during cell stress triggered by LRRK2. We do not see evidence of cell death in the inducible HEK 293FT cells used here, in contrast to results reported previously in human neuroblastoma lines or primary neurons. Therefore, 4E-BP and protein translation may be relevant to PD models, including those of Imai et al [18] and Tain et al [19] even if not directly through LRRK2 but rather through other stress induced protein kinases.

Of interest, Imai et al reported that 4E-BP could be co-immunoprecipitated with LRRK2, suggesting that the two proteins may form a complex rather than being a conventional kinase-substrate pair with high processivity. Perhaps supporting this idea, we did note that addition of 4E-BP to LRRK2 increased autophosphorylation of the kinase, which might feasibly occur via direct binding. In the case of LRRK2, 4E-BP phosphorylation can occur when complexed to eIF4E although at a slightly decreased rate. Previous studies [25] have shown that the phosphorylation of 4E-BP by MAP kinase is restricted when 4E-BP is bound to eIF4E. Phosphorylation of 4E-BP at Ser64 by an mTOR-associated kinase can result in dissociation of the 4E-BP/eIF4E complex [26]. Therefore, kinase activity towards 4E-BP, even at a single site can be influenced by, and influences, 4E-BP and eIF4E binding.

It is also important to note that these experiments do not prove that autophosphorylation of LRRK2 is an authentic physiological reaction. We and others, have recently mapped the autophosphorylation sites of LRRK2 to the ROC domain [27], [28] although these sites have not yet been proven to exist *in vivo*. It is equally likely that an as yet uncharacterized LRRK2 substrate may be more efficient than 4E-BP.

In summary, our data shows that *in vitro* LRRK2 mediated 4E-BP phosphorylation is weak compared to LRRK2 autophosphorylation. Additionally, the 4E-BP phosphorylation state in mammalian cells is unaffected by LRRK2 overexpression using a stable, inducible cell line approach or in transient transfections. These results allow us to conclude that 4E-BP is not a major substrate of LRRK2, and that there remains an urgent need to identify LRRK2 physiological substrates. Whether 4E-BP is a direct, authentic substrate of LRRK2 is unclear and should be considered provisional.

## Materials and Methods

### Chemicals and Reagents

Truncated recombinant LRRK2 wild-type and variants (LRRK2-G2019S and LRRK2-D1994A kinase dead) (aa 970–2527) were purchased from Invitrogen (Madison, WI, USA). Recombinant eIF4E protein was purchased from Origene (Rockville, MD, USA). Monoclonal anti-GST LRRK2 antibody and monoclonal anti-V5 LRRK2 antibody were purchased from Amersham (Piscataway, NJ, USA) and Invitrogen (Madison, WI, USA) respectively, and used at 1∶5000 for chemiluminescent immunoblotting. Antibodies against phospho 4E-BP (T37/46, T70 & S65) and non-phospho 4E-BP and eIF4E were purchased from Cell Signaling Technology (Danvers, MA, USA). [γ^33^P]ATP (10 mCi/ml) was purchased from Perkin Elmer (Waltham, MA, USA).

### SDS/PAGE and Western Blotting

For western blots, proteins were separated on 4–20% Tris-HCl gels (BioRad, Hercules, CA, USA) and were transferred onto PVDF Membrane (Millipore, Bedford, MA, USA). Membranes were blocked in 5% bovine serum albumin (BSA) in 1×TBS-0.1% Tween 20 buffer and incubated with the indicated primary antibodies overnight at 4°C or 2hrs at room temperature. Blots were washed and incubated with horseradish peroxidase conjugated secondary antibodies (Jackson ImmunoResearch, Iowa city, IA, USA) and developed by ECL (GE Healthcare, Piscataway, NJ, USA) and captured on BioMax film (Kodak, Rochester, NY, USA).

### Cell Lines

The tetracycline inducible HEK 293FT LRRK2 cell lines were generated using the Flp-In T-Rex system (Invitrogen). Flp-In T-Rex 293 host cells containing the tetracycline binding protein were purchased from Invitrogen and grown in medium containing DMEM, 10% (v/v) FBS, 2 mM L-glutamine, 1% (v/v) Pen-Strep, 100 µg mL^−1^ Zeocin. To introduce LRRK2 (wild-type, G2019S, R1441C) we modified pcDNA5/FRT/TO expression vector to accept Gateway cloning by ligating a attR lambda recombination signal using the topoisomerase cloning site, and confirmed by sequencing. LRRK2 (wild-type, G2019S, R1441C) was then introduced into the vector using the Gateway clonase (Invitrogen). The resulting plasmids were co-transfected with a p0G44 plasmid carrying the Flp-recombinase into the Flp-In T-Rex 293 host cells, and stable integrants were selected by resistance to 200 µg mL^−1^ hygromycin and 15 µg mL^−1^ blasticidin. The cells were then maintained in medium containing 100 µg mL^−1^ hygromycin and 15 µg mL^−1^ blasticidin.

### 4E-BP Expression and Purification

4E-BP1 cDNA (NM_004095.2) cloned in pReceiver-B01 vector was purchased from GeneCopoeia (Germantown, MD, USA). Recombinant protein were expressed in BL21(DE3) *Escherichia coli* (Novagen) in fusion with a non-cleavable N-terminal His_6_ tag (vector-derived sequence LEHHHHHH) for purification by metal affinity Ni^2+^-nitrilotriacetic acid chromatography. Bacteria were grown in Luria Bertani (LB) medium until reached an optical density (OD) of ∼0.6 and recombinant protein expression was induced by the addition of 0.5 mM of IPTG (isopropyl-thiogalactopyranoside). Cell pellets were stored at −80°C until needed. Cells were sonicated in lysis buffer (50 mM Tris-HCl, 50 mM NaCl, 0.5 mM EDTA, 5% glycerol, 0.1% Triton-X, 1 mM DTT and 1 mM protease inhibitor) and recombinant His_6_-tagged proteins purified using Ni^2+^-nitrilotriacetic acid His-Select resin (Sigma) and eluted with elution buffer (20 mM Tris, 200 mM NaCl and 200 mM imidazole). Purified 4E-BP ran as a single band of ∼20 kDa on Coomassie-stained SDS-PAGE and identity was confirmed by western blot with anti-4E-BP antibodies.

### *In Vitro* Kinase Assay

Non-radioactive *in vitro* kinase assays were set up in a total volume of 20 µl of kinase buffer [20 mM Tris/HCl (pH 7.5), 10 mM MgCl_2_, 1 mM EGTA, 1mM Na_3_VO_4_, 5 mM β-glycerophosphate, 0.02% Triton X-100, and 2 mM fresh dithiothreitol] containing ∼12 nM of truncated recombinant LRRK2 wild-type, LRRK2-G2019S or LRKK2 kinase dead with different concentrations of 4E-BP (1.5, 3, 6 and 12 µM) and ATP (100 µM). Reactions were stopped by the addition of SDS sample buffer and western blots were performed after electrophoresis of samples on 4–20% Tris-HCl gels. Blots were incubated with anti GST, phospho 4E-BP (T37/T46, S65 and T70) or total 4E-BP antibodies.

For kinetic studies and LRRK2 autophosphorylation experiments, truncated recombinant LRRK2 wild-type, LRRK2 kinase dead and LRRK2-G2019S were incubated separately with [γ^33^P]ATP (5 µCi/reaction) in kinase reaction buffer consisting of 20 mM Tris/HCl (pH 7.5), 10 mM MgCl_2_, 1 mM EGTA, 1mM Na_3_VO_4_, 5 mM β-glycerophosphate, 0.02% Triton X-100, and 2 mM fresh dithiothreitol at 30°C in a final volume of 20 µL at five different time intervals (0, 5, 15, 30 and 60 minutes) with four different concentrations of 4E-BP (1.5, 3, 6 and 12 µM). Reactions were terminated by the addition of SDS sample buffer (Invitrogen). LRRK2 autophosphorylation and 4E-BP phosphorylation were detected by autoradiography using a phosphor screen, scanned on a STORM 840 scanner (GE Healthcare, Piscataway, NJ, USA), and quantitated using IMAGE J software (NIH). The kinetic parameter K_m_ and V_max_ were determined by fitting the rate of reaction *versus* substrate concentration curves to the Michaelis-Menten equation, ν/V_max_ = [S]/([S]+K_m_) using a nonlinear regression analysis in Graphpad Prism software version 4.0 (GraphPad software, La Jolla, CA, USA). All fitted curves had R>0.9.

To estimate the effect of eIF4E on 4E-BP phosphorylation, LRRK2 wild-type (12 nM) were incubated separately with 4E-BP (3 µM), eIF4E (3 µM), 4E-BP and eIF4E complex (3 µM each) at five different time intervals. 4E-BP phosphorylation were detected by autoradiography using a phosphor screen. Blots were incubated with total 4E-BP and total eIF4E antibodies.

We also performed similar reactions with three other kinases. STK3 (serine/threonine kinase 3), MAPK14/p38α (mitogen-activated protein kinase 14) and DAPK2 (death associated protein kinase 2) were incubated separately with [γ^33^P]ATP (5 µCi/reaction) in kinase reaction buffer at 30°C in a final volume of 20 µL at five different intervals of time (0, 5, 15, 30 and 60 minutes) with 12 µM 4E-BP.

### Stochiometery of LRRK2 Autophosphorylation and 4E-BP Phosphorylation

*In vitro* phosphorylation assays performed as previously described, were run on SDS-PAGE and transferred onto PVDF membranes. After ponceau staining, bands corresponding to LRRK2 or 4E-BP were excised according to their molecular weight and incorporated radioactivity was assessed by LS 6500 mulitipurpose scinitillation counter (Beckman coulter, Sykesville, MD). A calibration curve was built with known amounts of radiolabeled ^33^P-ATP (equation: y = 2.49E-07×; R^2^ = 9.97E-01; where y = pmoles and x = cpm) and the number of moles of Pi (inorganic phosphates) incorporated per mole of protein was calculated by linear interpolation of the cpm (count per minute) data collected from the phosphorylated proteins.

### Mammalian Cell Expression Constructs

Full-length LRRK2, LRRK2-G2019S and LRRK2-R1441C were cloned to generate N-terminal 2×-myc as described previously [29], [30]. Human MAPK14/P38α gene was amplified from whole brain cDNA using primers 5′-ATGTCTCAGGAGAGCCCACGTTCTAC-3′ and 5′-TCAGGACTCCATCCTTCTTGGTCAAG-3′. MAPK14/P38α cDNA was subsequently cloned by gateway recombination from the donor vector pCR-GW8 (Invitrogen) into pCMV-2×Myc destination vector described by Greggio et al (29).

### Phosphorylation of 4E-BP in Inducible Cells and Transient Transfections

LRRK2 wild-type, LRRK2-R1441C and LRRK2-G2019S HEK 293FT inducible cells were grown in Dulbecco's modified Eagle's medium (Gibco, Carlsbad, CA) supplemented with 10% fetal bovine serum (Interon Inc., New York, NY) and penicillin (100 units/mL) and streptomycin (100 units/mL) at 37°C and 5% CO_2_. Cells were induced by 1 µg/µl doxycycline after one day in culture. Cells were harvested 24hrs after induction. 4E-BP phosphorylation was determined by western blot after electrophoresis of samples on 4–20% Tris-HCl gels. Blots were incubated with anti V5, phospho 4E-BP (T37/T46, S65 and T70) or total 4E-BP antibodies.

For transient transfection HEK 293FT cells were cultured as above and transfected using 40 ug of PEI (polyethyeleneimine) (Polysciences, Warrington, PA) for 20 ug of plasmid DNA. Cells were harvested 48hrs after transfection. 4E-BP phosphorylation was determined by western blot after electrophoresis of samples on 4–20% Tris-HCl gels. Blots were incubated with anti myc, phospho 4E-BP (T37/T46, S65 and T70) or total 4E-BP antibodies.
